# Retraction: Neural correlates of risky monetary decision-making impacting others

**DOI:** 10.3389/fnhum.2026.1972741

**Published:** 2026-08-25

**Authors:** Frontiers Editorial Office

Following publication, the authors contacted the Editorial Office, stating that during the fMRI processing procedure, the first 3 TRs were removed from the imaging data as part of standard slice timing correction, but the stimulus timing files were not adjusted accordingly, resulting in error. The conclusions reported in the article are therefore no longer supported by the analyses. An investigation was conducted in in accordance with Frontiers' policies that confirmed this; therefore, the article has been retracted.

The authors are invited to submit a new manuscript for peer review once these concerns have been addressed.

This retraction was approved by the Chief Editors of Frontiers in Human Neuroscience and the Chief Executive Editor of Frontiers. The authors agree to this retraction.

